# Applications of Extended Platelet Profiles in Clinical Practice

**DOI:** 10.3390/diseases14040116

**Published:** 2026-03-25

**Authors:** Yi Yuan Zhou, Robert W. Maitta

**Affiliations:** Department of Pathology, University Hospitals Cleveland Medical Center, Case Western Reserve University, Cleveland, OH 44106, USA; yiyuan.zhou@uhhospitals.org

**Keywords:** platelet profile, thrombocytopenia, immature platelet fraction, absolute immature platelet count, sepsis, ITP, TTP, SLE, inflammation

## Abstract

Thrombocytopenia is a frequent complication of patients presenting emergently across the world for a wide array of etiologies. From patients who develop thrombocytopenia due to invasive neoplastic disease affecting the bone marrow to patients who develop immune complications secondary to the formation of auto-antibody responses that drive patients’ platelet counts lower or even cause infection, these patients stress the clearest need for prompt tests to discern the more likely thrombocytopenic-inducing cause. It is in this setting that looking at other platelet variables easily obtainable from modern hematology analyzers has gained traction. One of the elements found in extended platelet profiles are immature platelets (youngest and newly released platelets), also known as reticulated platelets, which are readily measurable from a complete blood count. One of the advantages of obtaining these counts is that they represent the immediate response of the bone marrow to the thrombocytopenia and, depending on etiology inducing the thrombocytopenia, they also provide information on the marrow’s response to therapeutic approaches. It is in this context that this review will present information of how these relatively novel platelet parameters can be used in clinical practice and how they can be a rapid gauge of the body’s response to disease processes leading to platelet losses. Thrombocytopenias resulting from infection (sepsis, viremia), autoantibody formation (immune thrombocytopenia and immune-mediated thrombotic thrombocytopenic purpura), immune dysregulation (systemic lupus erythematosus), and iatrogenic (drug-induced) will be discussed and used to explain how these young platelet measurements can provide valuable clinical information.

## 1. Introduction

Diseases that lead to thrombocytopenia not only cause changes to platelet counts in circulation, but also trigger a sequence of events resulting in either higher or decreased output of new platelets from the bone marrow. Some of these etiologies can be immunologic affecting platelets either directly or indirectly, iatrogenic, or secondary to mechanistic deficiencies in the bone marrow itself. Etiologies affecting new platelet production, and especially platelet counts, represent clinical challenges not only for diagnosis but also for treatment. These etiologies at times cannot be addressed by just transfusing more platelets, since the disease can be made worse by transfusion. Thus, prompt laboratory testing that determines the type of thrombocytopenia affecting the patient is of the utmost importance.

### 1.1. Thrombocytopenic Etiologies

Nowadays there is a better understanding of diseases caused by immune dysregulation leading to autoantibody formation to either platelets, platelet mediators or to molecules needed for proper platelet function. Thrombocytopenia can be segregated into three “severity categories”. A patient with a platelet count of 100–150 × 10^9^/L is considered mild, moderate is considered as patients with counts 50–100 × 10^9^/L, and severe is reserved for patients with counts < 50 × 10^9^/L [1]. Diagnostically, however, there are still challenges differentiating etiologies since thrombocytopenic presentations can have overlapping clinical pictures that make discerning a particular entity difficult [2]. An example of this is malignant conditions with different degrees of thrombocytopenia that are confused with immune thrombocytopenia (ITP) [3]. For this reason, biomarkers that improve diagnostic capabilities in a time-efficient manner are needed. One type of functional biomarker that has gained interest recently is the formation of Neutrophil extracellular traps (NET) in response to infection; however, this is not a laboratory test, is not derived from a complete blood count (CBC), and it has recently been comprehensively reviewed by others [4,5]. An ever-expanding literature has developed over the last two decades outlining the benefits of obtaining a more complete platelet profile to differentiate thrombocytopenic presentations. One of these approaches, is looking at the immature platelet fraction (IPF) also known as reticulated platelet percentage, which represents the youngest platelets found in circulation, recently released from the bone marrow, and reported by modern automated hematology analyzers with fluorescence capabilities [6,7,8,9,10,11]. From IPF, other variables can be derived such as the absolute immature platelet count (A-IPC). These counts have been used across thrombocytopenic states to discern an etiology providing timely information in clinical settings [6,7,8,9,10,11,12].

### 1.2. Immature Platelet Production

Immature platelets are significantly larger, with higher RNA content, and more biochemically active than mature platelets [13]. As a result, they have greater sensitivity to chemotherapy and irradiation, and can be found elevated above reference ranges in response to immune conditions targeting platelets [13,14]. Notably, immature platelets are stable in a specimen even 24 h after phlebotomy and counts can still be obtained within this time frame [13,15]. In consumptive thrombocytopenic processes and those due to platelet hypoproduction, low A-IPCs are a frequent finding [16]. These low immature platelet counts often suggest that a given etiology has an inhibitory effect over the bone marrow [8,17]. Importantly, A-IPCs are reported not to be influenced by neither gender [18] nor age since their production is maintained as we age [19]. For example, among maternal thrombocytopenia predictors of a favorable response to intravenous immunoglobulin (IVIG) therapy is an IPF of 16% [20].

Immature platelets are measured from a CBC, emphasizing that obtaining extended platelet profiles that include A-IPC does not delay timely results [21]. As hematology analyzers become more advanced, analytical times have been further reduced. Improvements in dyes, gating adjustments to improve specificity, size correction, and wavelength detection have made obtaining counts fast and highly reproducible [22,23,24]. These improvements have allowed for the development of protocols that sort and isolate immature platelets making it possible for research to be carried out on these platelets [25,26]. Importantly, preclinical variables such as time from collection, extent of platelet activation, anticoagulant used, and matrix elements such as degree of hemolysis do not necessarily impede immature platelet testing [22,27,28,29,30]. However, their application requires establishment of reference intervals for results interpretation [22,23].

It has been reported that A-IPC changes precede mature platelet count changes by 2–3 days [31,32,33]. One of these patient populations showing that immature platelets are a useful biomarker is hematopoietic stem cell transplant recipients, who show increases in A-IPC that foretell platelet count recovery and thus engraftment [31,34,35]. On the other hand, patients with bone marrow failure or invasive disease that involves the bone marrow experiencing low A-IPCs do not compensate for existing thrombocytopenia [6]. Thus, this review will present the current literature describing how immature platelets can enhance the information conveyed by a CBC and how they are being used as earlier biomarkers to diagnose and treat thrombocytopenia-inducing etiologies.

## 2. Immature Platelets in Sepsis

Besides being described as mediators of hemostasis, platelets are bona fide members of the immune system since through their responses to pathogens either via phagocytosis or interactions with most cells of the immune system through secreted mediators and direct cell–cell interactions they orchestrate responses to infections [1]. Sepsis is characterized by dysregulated host response to infections resulting in organ dysfunction with potentially life-threatening consequences [36,37]. Sepsis is one of the most frequent causes of death worldwide, and it has been estimated that the global annual sepsis incidence is around 276–678/100,000 persons with case fatalities ranging from 22.5 to 26.7% [38]. Multiple biomarkers exist for detection of sepsis that allow for earlier intervention to reduce morbidity and mortality. In this regard, lactate (LA), C-reactive protein (CRP), and procalcitonin (PCT) are some of the most commonly used biomarkers for sepsis; however, these are not specific to sepsis and research is ongoing to identify additional markers to improve early sepsis detection [39,40,41].

Activation of coagulation has been implicated in the pathogenesis of severe sepsis and thrombocytopenia has been linked to poor prognosis in critical illness [42,43]. Since IPF is a measure of immature platelets that often increases prior to thrombocytopenia, this has motivated research into exploring its use as an easily accessible biomarker for sepsis [9]. Multiple studies, involving primarily intensive care unit (ICU) patients in both adult and pediatric settings, have reported significantly higher IPF values in patients with sepsis [44,45,46]. In an observational study of ICU patients over a 7-day period, it was found that there was an increase in mean IPF for both patients with sepsis on admission (4.1%) and patients who developed sepsis within the observational period (5.3%) when compared to patients without sepsis (3.0%) [44]. Importantly, significantly higher IPF was also noted in patients 2–3 days before the diagnosis was made suggesting a potential use of IPF as an early maker for predicting the development of sepsis. These findings were corroborated by a separate study that looked at A-IPC and quantified the risk of developing sepsis in the ICU as a 13% increase in risk for every unit increase in A-IPC [45]. In the pediatric ICU (PICU) setting, a higher IPF in patients with sepsis has been reported as well; however, in these cases, IPF showed only moderate performance in differentiating septic from non-septic patients with an area under the curve (AUC) of 0.642 compared to the AUC of CRP (0.796) and PCT (0.828) [46]. It is important to note that at the optimal IPF cut off of >2.7%, its positive predictive value, negative predictive value, and accuracy were on par with PCT, the best performing biomarker, suggesting a possible influence of sepsis prevalence on diagnostic performance. While the IPF observed in many studies were statistically significant, there was considerable overlap in values between patients with and without sepsis, which may partially explain differences in IPF diagnostic performance. On the whole despite reported differences, IPF appears to be useful in identifying ongoing sepsis and is comparable to established biomarkers such as CRP, PCT, LA, and immature granulocytes. A summary of the measured mean or median IPF is presented in Table 1.

Detection of bacteremia is one of the key criteria of sepsis, but traditional culture can take 2–4 days, delaying targeted intervention [54]. An early study involving 153 adults reported that blood culture positive samples had a higher mean IPF of 4.86% compared to a mean of 1.79% in those who were culture negative (Table 1) [47]. This finding is supported by a clinical study of late preterm neonates with respiratory distress that demonstrated higher IPF in patients with infectious causes vs. those without [49]. Using an IPF cutoff of >2.9% measured 12–24 h after birth, the authors concluded that IPF moderately predicted pneumonia with a sensitivity of 65% and specificity of 71.4%. This correlation between infection and higher IPF was also noted in adult populations [51]. Furthermore, the observation that IPF dropped within 48–72 h after initiating antimicrobial therapy, further strengthened the linkage between IPF and bacteremia, while lending support to its use as an early predictor of culture positivity and antimicrobial response [49].

For earlier detection of sepsis, there has been growing interest in determining if there is a relationship of IPF to sepsis severity. In one small study comparing IPF of 11 patients with sepsis and 12 patients with severe sepsis or septic shock as defined by sepsis severity scores, the mean IPF was significantly higher in patients with severe sepsis or septic shock (6.2%) compared to less severe form of sepsis (3.6%) [48]. This was corroborated in a larger study where a significantly higher median IPF was found in patients with complicated (5.3%) vs. uncomplicated sepsis (4.1%), both of which were in turn higher than the IPF of patients without sepsis (2.9%) [52]. By contrast, while authors concluded that IPF can discriminate between septic and non-septic patients, it performed less well at differentiating between uncomplicated and complicated sepsis, having only moderate sensitivity of 64.9% and specificity of 53.1% when using the optimal cutoff IPF of >4.1%. Concurrently, in an ICU study of septic patients, higher IPF (4.3% median compared to 2.1% in controls) was identified 1–5 days prior to a fall in platelets, signaling severe sepsis [55]. Multivariate cox regression revealed that IPF was an independent predictor marker for 28-day mortality with an accuracy rate on par with the sepsis severity score, acute physiology and chronic health evaluation II (APACHE II) (AUC 0.886 for IPF vs. 0.857 for APACHE II). Furthermore, the authors noted that a combination of IPF and APACHE II score increased the AUC of 28-day mortality to 0.912. By contrast other variables such as CRP, PCT, and coagulation markers showed either a low AUC or did not reach statistical significance.

Thus far, the discrepancies in predictive performance of IPF for sepsis can be attributed to different measurements, distinct hematology analyzers, and individual variations in values. As a consequence, other studies moved away from singular IPF measurements towards a measurement of change in IPF (ΔIPF) to minimize individual variations [53,56]. While these studies confirmed ΔIPF to be significantly higher in patients with sepsis or bacteremia, there were persistent differences in the performance of ΔIPF prediction of sepsis. One of these studies indicated that ΔIPF from day 1 to day 2 of ICU stay provided a strong predictor for sepsis with an AUC of 0.9113, vastly higher than CRP or PCT whose AUCs were 0.6233 and 0.6579 respectively [56]. At the optimum cutoff ΔIPF of 1.95% the authors noted a relatively strong sensitivity of 75.0% and an excellent specificity of 96%. Importantly, they reported that a combination of ΔIPF and day 2 PCT, provided a positive predictive value and negative predictive value of 100% and 96.1%, respectively. In the second study, they defined ΔIPF as the difference in IPFs between the day the blood culture sample was collected and the IPF from the day prior [53]. A ΔIPF cutoff of >3.4% conferred higher specificity of 97.3% in predicting bacteremia but with low sensitivity of 25.3%. Similar to the prior study, combination of both ΔIPF and PCT led to improved diagnosis resulting in excellent sensitivity of 90.5% and a moderate specificity of 56.6%. When they looked at the 30-day mortality in relation to ΔIPF within the bacteremia group, they found a higher value in those who did not survive (2.7% in non-survivors compared to 0.8% in survivors); the 30-day mortality prognosis of ΔIPF at a cut off of >1.5% showed similar prognostic performance to PCT.

Infections result in diffuse consumption of mature platelets as the body fights off the offending infectious agent(s). However, despite the developing thrombocytopenia being a sign in these clinical settings, A-IPCs are maintained so that production of new immature platelets by the bone marrow tries to keep pace with the increased platelet turnover [57]. Notably, A-IPC increases appear to be associated with a higher mortality risk and represent a greater degree of disease severity [58]. These A-IPC increases occur earlier in the disease course prior to sepsis onset [45], and this change in immature platelets predicts ensuing mature platelet count decreases once infection sets in [55]. This could be a direct result of higher immune hyperreactivity during severe infection that results in disseminated platelet consumption with declining mature platelet counts; thus, requiring a higher immature platelet output. Such responses however, may be limited to adult patients since neonates do not demonstrate such increases, instead being characterized by suppressed A-IPC that is associated with disseminated infections and poor survival [59]. Along these lines, older children who recovered from dengue fever had increased immature platelet outputs up to 3 days prior to recovering their platelet count [60]. Therefore, in infections, the negative feedback between immature platelets and mature platelets appears preserved in older children and adults contrary to neonates.

**Figure 1:**
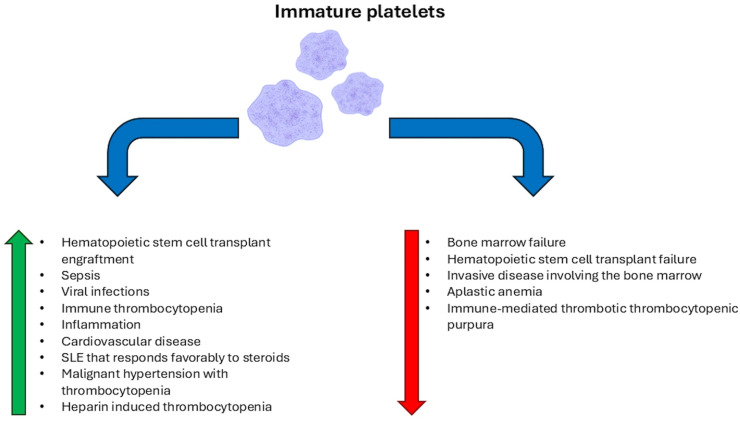
Immature platelet count changes in association with diseases. Etiologies in which IPF/A-IPC is increased are indicated by the green arrow and those where IPF is reported to be decreased are indicated by the red arrow.

In summary, studies indicate a significant correlation between increased IPF and A-IPC in patients with sepsis or who are at risk of worsening disease (Figure 1). Unlike, other markers, immature platelets help predict sepsis severity. Likewise, compared to markers such as CRP or PCT, immature platelets can be performed off of a CBC, making it an attractive sepsis biomarker. Despite these advantages, there is currently no consensus in the optimal cutoff value of IPF or when and how often IPF should be measured, which explains the differences in IPF performance in predicting sepsis. Furthermore, while significant differences were noted in mean IPF in sepsis compared to non-sepsis, IPF values had a wide range with overlap between the two groups that further complicated test performance. Nevertheless, even though it remains unclear if immature platelets are sufficient as a standalone biomarker, integration of IPF in the laboratory workup of sepsis along with traditional biomarkers contribute towards earlier sepsis detection.

## 3. Immature Platelet Count Changes in Viral Infections

During the recent pandemic it soon became apparent that patients with difficulty overcoming COVID-19 infection had thrombophilia as a sign of severe disease. COVID-19 patients who were severely ill developed anemia, lymphopenia, and thrombocytopenia in a background of significantly higher IPF and H-IPF (highly fluorescent immature platelet fraction) upon hospital admission [61]. The latter are the newest among immature platelets released from bone marrow. Looking at patients more closely, it was reported that elevated IPF was associated with increased inflammatory and thrombotic activity in male hospitalized COVID-19 patients under 70 years of age with moderate-to-severe disease [62]. An analysis of a large adult cohort of over 600 COVID-19 patients indicated that an increased IPF at presentation predicted greater length of hospitalization and ICU admission; and this was of greater magnitude when comparing ICU to non-ICU patients (6.9 + 5.1 vs. 5.3 + 8.4, *p* < 0.01), and deceased patients to those who recovered (7.9 + 6.3 vs. 5.4 + 7.8, *p* < 0.01) [63]. Likewise, peak A-IPC was significantly higher comparing pediatric ICU to non-ICU patients (18.5 + 16.2 vs. 13.2 + 8.3, *p* < 0.05), and between patients requiring ventilator support than those without (22.1 + 20.1 vs. 13.4 + 8.4, *p* < 0.05) which indicated that high initial and peak values of IPF and A-IPC represented prognostic biomarkers for COVID-19 progression to severe disease (Figure 1) [63]. Specifically, high immature platelet counts predicted those pediatric patients needing ICU admission and requiring mechanical ventilation [64]. In another report, increases in A-IPC occurred as early as three days after admission in COVID-19 patients compared to patients with sepsis (*p* = 0.007) [65]. Taken together, these findings suggest that immature platelets can be biomarkers for disease severity in COVID-19 patients.

Thrombocytopenia is also found in infections with other viral pathogens. For example, thrombocytopenia is a common and possibly serious finding in dengue virus (DENV) infection where platelet depletion is critical, and results in severe dengue symptoms since this infection impairs both megakaryopoiesis and thrombopoiesis [66]. Likewise, platelet elimination via platelet activation, enhanced apoptosis, and clearance during infection are elevated in DENV infection resulting in increased immature platelet production as disease progresses [66]. In a study of almost 300 patients with confirmed DENV infection, a decrease in platelet count in the first week of illness was observed, with a concomitant increase in IPF more than 3 days after fever onset and prior to platelet recovery [67,68]. In pediatric DENV infection patients (36 with dengue fever and 28 with hemorrhagic fever) reported an IPF of ≥10.0% after defervescence (normal children was 3.6%) and this predicted platelet recovery to a hemostatic count of ≥60 × 10^9^/L within 72 h [60]. In a similar study of 32 patients with dengue fever, IPF correlated strongly with platelet count recovery so that a peak IPF was preceded by a 24–48 h platelet count improvement if IPF was more than 10% [69]. Finally, a study of over 600 patients reported that IPF of dengue patients was twice that of individuals with bacterial infection (3.7% vs. 1.9%; *p* < 0.001) which increased further during the critical phase of disease (5.2% vs. 2.2%; *p* < 0.001) [70]. Thus, IPF can be an early recovery biomarker in patients with dengue and thrombocytopenia.

Changes to IPF can also be seen in patients with Crimean–Congo hemorrhagic fever (CCHF). Such patients have higher IPF compared to controls and these values are positively correlated with duration of hospital stay [71]. The receiver-operating-characteristic analysis suggested that IPF > 10.5% aided in CCHF diagnosis in patients with positive radiological findings, suggesting that routine immature platelet measurement is useful to follow-up disease course [71]. Likewise, an analysis of over 150 patients with chronic hepatitis B indicated that in patients who developed thrombocytopenia due to infection, IPF was significantly increased compared to non-thrombocytopenic patients and healthy controls (both *p* < 0.001) [72]. This suggests that thrombocytopenia during hepatitis B caused by destruction/sequestration of platelets in the spleen drive corresponding increases in IPF [72]. In human immunodeficiency virus (HIV)^+^ patients with thrombocytopenia associated with different etiologies, IPF responses were dependent upon the integrity of the bone marrow. For example, the majority of patients with ITP had high IPF while the significant majority of patients with hypocellular marrow had decreased IPF that was only 1/3 of that observed in ITP subjects. IPF responses were variable in patients with malignant marrow infiltration or mycobacterial invasion [73]. Unexpectedly, a significant positive association of IPF responses with HIV viral load was encountered. Therefore, it can be concluded that IPF responses are dependent upon the degree of thrombocytopenia and the ability of the bone marrow to respond proportionately to platelet count decreases.

## 4. Immature Platelet Count Changes in ITP

ITP is a clinical syndrome involving extensive platelet destruction secondary to autoantibody production, oxidative stress, and limited megakaryopoiesis [74]. The latter becomes important when considering IPF as a diagnostic biomarker. It has been two decades since a report indicated that increases in IPF from its baseline was evident in patients with ITP during acute presentations or disease exacerbations, which was indicative of bone marrow response to the thrombocytopenia [75]. This results in a higher platelet distribution width due to the higher proportion of large young immature platelets in circulation [76]. In this disease, the degree of platelet destruction and resulting thrombocytopenia appears to determine the higher risk of associated bleeding [77]. In ITP, IPF tends to be significantly higher compared to controls and patients with bone marrow failure, specifically ≥25% of platelets in circulation are immature and this increase is an independent predictor of ITP diagnosis [78]. This, however, becomes of greater significance when A-IPC changes are analyzed. An A-IPC of 2.1 × 10^9^/L or better has been suggested to differentiate ITP from aplastic anemia [79]. As a result, an algorithm that takes isolated thrombocytopenia without evidence of dysplasia in peripheral blood, normal-to-high thrombopoietin, and IPF at the upper limit of normal to suspect ITP has been developed [80]. Furthermore, lower platelet counts uniformly result in much higher IPF (27%) in new ITP patients compared to patients with higher platelet counts at presentation [81].

It has been reported that changes in A-IPC characterize ITP patients responding to treatment [11,82]. Pre-treatment A-IPC appears specific and indicative of treatment response to dexamethasone in ITP when compared to other variables measured [83]. On the contrary, variables such as platelet sialylation have failed to prove utility in disease diagnosis and management since it does not differ between patients with ITP and those with inherited forms of thrombocytopenia (IT), even though platelets are in a higher activated state in ITP compared to IT [84]. Recently, a study suggested that pseudothrombocytopenia is associated with ITP, so it is necessary to differentiate them which can be done based on presence of larger platelets (immature) and higher IPF compared to controls [85].

Cumulative evidence indicates that during ITP presentations, the bone marrow attempts to compensate for platelet destruction by increasing A-IPC output [3,11,29,75,86,87,88]. However, there are measurable differences in the degree of compensation between patients with chronic ITP vs. those with new onset disease. Immature platelet output is higher in chronic ITP patients [89], especially among those patients with a higher bleeding risk [86,90,91]. These bone marrow compensatory increases in ITP patients are even more significant when taking A-IPC changes into account [92]. For example, a greater specificity in predicting bleeding risk is indicated by a significantly low A-IPC [90]. Nevertheless, an isolated study which raised concerns that immature platelet measurement was of limited use in differentiating ITP from other etiologies, acknowledged that this was likely due to the type of hematology analyzer used but did not provide A-IPC data [93].

In regard to being used as a biomarker to develop a diagnostic prediction scoring model, an IPF of at least 7% characterizes patients that is more predictive compared to mature platelet counts [3]. When combined with red blood cell counts, hemoglobin concentration and lymphocyte count, IPF significantly improved the specificity and sensitivity of scoring models [94]. In children, scoring models and development of markers are similarly needed to aid in triaging and increasing suspicion of disease. Reports describing that pediatric ITP patients can be readily identified by an IPF of 9.4% from other patient populations, and that this threshold predicts prognosis and remission once immature platelets return to baseline are encouraging [89]. A-IPC changes have shown a high positive predictive value [95], with the added benefit that higher immature platelet counts are preceded by >2 days of corresponding quantitative changes in mature platelet counts [33]. This supports development of a disease model based on immature platelet counts in which their quantitative relation to mature platelet counts through a negative feedback mechanism is explained. Thus, in ITP, when platelets get consumed or destroyed in the periphery due to the disease process, the bone marrow responds by increasing immature platelet output to compensate for losses [14]. Once mature platelet counts improve in therapy and remission is achieved immature platelet counts return to baseline [14,96].

## 5. Immature Platelet Count Changes in Thrombotic Thrombocytopenic Purpura (TTP)

TTP is a severe type of microangiopathic hemolytic anemia (MAHA) that historically led to significant morbidity and mortality due to the formation of diffuse microthrombi affecting organ systems throughout the body [97]. Deficiency of the ADAMTS13 enzyme defines the disease and this can be either innate (congenital) or immune-mediated (i) secondary to the presence of the antibody to the enzyme [98,99]. To differentiate the two forms, anti-ADAMTS13 antibody would be absent in congenital TTP but detectable in iTTP [100,101]. The antibody formed can be either neutralizing or not depending if the antibody is specific to the spacer domain of the ADAMTS13 molecule [102]. The gold standard test is a measurement of ADAMTS13 activity using the fluorescence resonance energy transfer test (FRET) which measures cleavage products of a fluorescently labeled synthetic vWF peptide, with an activity of <10% considered as diagnostic [103]. This test, however, is labor-intensive and not available at most institutions, which have to send it out to large reference laboratories. This explains why daily therapeutic plasma exchange (TPE), using plasma as replacement fluid, which is first-line therapy is often initiated empirically as soon as the disease is suspected despite ADAMTS13 results not being available. This is not without potential risks, since TPE using plasma is associated with adverse events such as transfusion reactions and apheresis-associated complications making a timely accurate diagnosis essential [104,105,106]

Accordingly, alternative test approaches that increase the suspicion for TTP without delaying therapy initiation can be clinically useful. Twenty years ago it was described that the IPF of iTTP patients was significantly lower than ITP patients [75]. Reports from our group have shown that patients who were later found to have ADAMTS13 activity consistent with iTTP had A-IPCs at presentation significantly lower and below the reference range, than healthy controls and other thrombocytopenic patients without enzyme deficiency [32,107,108]. In patients with refractory disease, A-IPC obtained early in the disease course was useful in establishing therapy needing adjustment [109,110]. In iTTP patients, improvement in A-IPC uniformly preceded by 2 days corresponding changes in mature platelet counts following initiation of daily TPE [32,107,111]; and A-IPC returned to baseline once mature platelet counts normalized [111]. Regardless, the most significant finding was that A-IPC suppression at presentation was seen in all patients who were found to have iTTP, that these counts had strong correlation with ADAMTS13 activity < 10%, and that this ruled out most patients who had other MAHA etiologies [112]. Interestingly, A-IPC suppression or decrease in counts appeared to be not as severe in patients with relapsing disease, and the magnitude of the A-IPC decrease appeared dependent upon the mature platelet count [112,113]. A-IPC also predicted response to TPE in patients with high ADAMTS13 inhibitors who, for the most part, required a greater number of procedures to restore platelet counts [111,114]. A-IPC measurement had the added benefit that it identified iTTP or ruled it out even in cases where the PLASMIC score, which has been proposed to increase suspicion of a TTP diagnosis, underestimated or overestimated presence of disease [114]. Furthermore, unlike the PLASMIC score which has been shown not to be useful in pediatric settings, A-IPC specifically identified children with iTTP even among those whom the score suggested disease was not present [114,115].

Etiologies such as pseudo-thrombotic microangiopathy represent one of those MAHA-like presentations that can be confused with iTTP. It is a rare complication of B12 deficiency that can be readily identified by testing for the presence of intrinsic factor antibodies in the setting of normal B12 levels [116]. Even though it is unclear what the immature platelet counts are in such settings due to its rarity, it is worth mentioning that testing for this entity may reveal if similarities or differences exist with A-IPC seen in iTTP. Case in point, in pregnant patients, IPF testing in combination with schistocyte counts readily discerns TTP from hemolysis, elevated liver enzymes, and low platelet count (HELLP) syndrome [117].

Suppressed A-IPC of iTTP patients at presentation suggest either disruption or suppression of the platelet production’s negative feedback that is rapidly reversed by initiation of daily TPE [32,107,111]. This finding favors the presence of additional “central” pathological insults needed for iTTP presentation to occur [101]. For example, using the zebrafish ADAMTS13 knockout model, it has been shown that just as in humans there is a higher number of vWF multimers characterized by a marked decrease in the number of immature and mature thrombocytes in a background of erythrocyte fragmentation and inflammation [118]. Based on these observations, a model in which impaired immature platelet/mature platelet negative feedback is part of the pathophysiology of new onset iTTP can be discerned [101]. In this disease model, the bone marrow does not respond uniformly to the existing thrombocytopenia with a corresponding increase in immature platelets unless TPE is initiated. Once apheresis is started, A-IPC steadily increases prior to the equivalent change in mature platelet count, and this results in restoration of the platelet production negative feedback. Finally, when mature platelet counts reach a normal nadir, A-IPC returns back to baseline. Future research addressing the mechanism(s) underlying A-IPC suppression and disruption of the negative feedback in iTTP patients is needed.

## 6. Immature Platelets in Inflammatory Settings

Inflammation-inducing disease processes that result in impaired thrombopoiesis can be triaged looking at immature platelets as shown in patients with impaired liver function/cirrhosis [119]. Similarly, states in which inflammation leads to platelet count changes can be discerned looking at immature platelets. For instance, high immature platelets in circulation predict those patients at risk of developing subsequent inflammation post cardiac surgery [120]. Even a week after surgery, a correlation between pro-inflammatory interleukin (IL)-6 in patients and immature platelet counts has been reported [121]. This is because increases in immature platelets can be stimulated by IL-6, since this cytokine drives thrombocytosis and platelet activation in a setting of intestinal inflammation [122]. However, this higher risk to cardiac patients associated with higher immature platelets may be related to the disease itself since HIV patients on antiretroviral therapy with cardiovascular disease have significantly higher immature platelets compared to HIV patients without cardiovascular disease on same therapy [123].

Thrombocytopenia can be seen in patients with systemic lupus erythematosus (SLE), but this tends to be a rare complication of the disease accounting for only 3–10% of cases. For example, in a patient with SLE who received steroids for over a decade to treat SLE-induced autoimmune hemolytic anemia, thrombocytopenia manifested as nasal bleeding, petechiae, and purpura with findings suggestive of ITP [124]. Laboratory examination revealed that thrombocytopenia presented with hypocomplementemia, and positive anti-cardiolipin and anti-β_2_-glycoprotein I IgG antibodies. Steroid pulse therapy, followed by high dose prednisolone and hydroxychloroquine on alternate days resulted in an increase in platelet count with concomitant decreases in IPF from 14.9% to 6.3%. Anti-cardiolipin and anti-β_2_-GPI antibodies, considered to be associated with thrombocytopenia and higher risk of thrombotic events, led to aspirin use after platelet count normalization to prevent thrombosis [124]. Likewise, immature platelets appear to correlate with SLE disease activity. In a large cohort of 282 SLE patients, it was shown that 12.4% of patients had thrombocytopenia [125]. Importantly, even though IPF correlated with platelet count, A-IPC was a significantly better marker in relation to the disease activity index. Both, the disease severity index and thrombocytopenia were independent parameters that accounted for immature platelet increases. The probability of clinical remission in A-IPC-high patients was higher than in A-IPC-low patients, indicating that as a biomarker it is better at predicting response to steroids in thrombocytopenic patients with SLE [125].

Inflammation is associated with hypertension and ensuing cardiovascular disease [126]. Malignant hypertension patients who developed thrombocytopenia have been reported to have significantly higher immature platelet counts that differentiates them from patients with MAHA processes such as TTP [108,127]. Likely, the shear forces experienced in hypertension damage the vasculature causing platelet consumption driving higher immature platelet output from the bone marrow. Similarly, as reported in other inflammatory presentations, it appears that A-IPC differentiates preeclampsia from patients with HELLP syndrome [127]. Smoking represents an inflammatory insult that causes vascular stenosis and inflammation resulting in hypertensive-like changes and higher concentration of immature platelets [128]. Interestingly, states of low-grade inflammation do not appear to provide a sufficient stimulus to drive immature platelet production [129]. Without question, additional research is required to further understand how immature platelet count changes play in inflammatory presentations.

## 7. Immature Platelets and Drug-Induced Presentations

Immature platelets have been used to establish when drugs affect thrombopoiesis [130]. In heparin-induced thrombocytopenia (HIT), antibody-mediated reactions to complexes that include platelet factor 4 are driven by heparin use. Since platelets are removed from circulation by the presence of antibodies to PF4-heparin complexes, changes in immature platelet output occurs. Increases in IPF have been reported in samples tested during HIT investigations [131]. Of interest, patients testing positive (HIT^+^) for the presence of anti-PF4-heparin antibodies have A-IPC similar to the reference range unlike patients who tested negative and had immature platelets well below this level [132]. This appears to indicate that HIT^+^ patients have immature platelet responses that attempt to maintain platelet count not necessarily in sufficient amounts. These results indicate that further research to elucidate the mechanisms by which the body, and specifically the bone marrow, compensates in the setting of drugs leading to thrombocytopenia are needed.

## 8. Conclusions

This review has presented a growing body of evidence reporting the use of immature platelet measurements in a variety of thrombocytopenic settings. Studies in the presented clinical areas are not all inclusive since new reports are frequently published showcasing the use of these counts. These studies support the development of clinical trials looking at how immature platelet counts change during disease presentation and how they provide clinical information that affects therapy timing. This greater understanding of immature platelets will depend on deriving adequate and updated-as-needed reference ranges to minimize discordant results, since newer analyzers are becoming more specific and sensitive. Therefore, expanding the discerning potential of CBCs by obtaining immature platelet counts unlocks a better understanding of thrombocytopenic-eliciting etiologies. Finally, immature platelet counts are equivalent to reticulocyte counts, which are routinely obtained in anemia presentations, but have a major advantage in that they represent real-time thrombopoietic output indicative of bone marrow response to disease and response to therapy initiation. Future studies looking at immature platelets, including clinical trials, will result in increased awareness of methodology, diagnostic potential, and higher usage in thrombocytopenic presentations.

## Figures and Tables

**Table 1:** Summary of studies and their reported IPF in control groups and sepsis/sepsis-related conditions. IPF values are expressed as either mean ± standard deviation or median (interquartile range (IQR)) or median (range) as indicated.

Study	Study Population	No Sepsis/Sepsis Related Condition IPF	Sepsis/Sepsis Related Condition IPF	Optimal Cutoff Value for IPF for Diagnosing Sepsis
[45]	ICU	Median 4.7 (3.2–7.1) IQR	Median 6.3 (4.8–9.5) IQR	n/a
	21 developed sepsis during study			
	41 without sepsis			
			
[44]	Adult ICU	Mean 3.0 ± 1.6	Mean 5.2 ± 2.6 in those who developed sepsis during observation period	>4.7%
	31 with sepsis		Mean 4.1 ± 2.5 in those with sepsis	
	33 developed sepsis during observation period			
	31 without sepsis			
[47]	Adult patients	Mean 1.79 ± 0.63	Mean 4.86 ± 2.67	n/a
	153 blood samples for culture			
[48]	Adult ICU	N/A	Mean 3.6 ± 2.6 for patients with sepsis	n/a
	11 with sepsis		Mena 6.2 ± 3.0 for patients with severe sepsis/septic shock	
	12 with severe sepsis/septic shock			
[49]	NICU	Mean 2.93 ± 0.75	Mean 4.62 ± 2.53	>2.9%
	16 with transient tachypnea of newborn			
	14 with congenital pneumonia			
[50]	NICU	Median 3.7 (0.9–6.5) range	Median 9.2 (4.4–39.2) in early onset sepsis	>5.5% in early onset sepsis
	50 early onset sepsis		Median 14.6 (6.1–32.3) in late onset sepsis	>6% in late onset sepsis
	56 late onset sepsis			
	44 control patients			
[51]	Adult	Mean 1.72 ± 0.77	Mean 2.76 ± 2.27	n/a
	45 patients with lower respiratory tract infection			
	39 healthy patients			
[52]	Adult	Median 2.9 (1.1–5.8) range for non-septic patients	Median 4.1 (0.8–25.6) range for uncomplicated sepsis	>4.1
	215 septic patients with 64 complicated sepsis and 151 complicated sepsis	Median 3.2 (1.1–11.3) range for non-septic patients with local infections	Median 5.3 (0.8–37.4) range for complicated sepsis	
	97 non-septic patients separated into non-septic and non-septic with local infection			
[53]	Adult admissions	ΔIPF of 0.7 (8–39) IQR	ΔIPF of 1 (0.4–3.6) IQR	>3.4
	75 with bacteremia			
	75 without bacteremia			
[46]	PICU	Median 0.85 (0.56–1.3) IQR	Median 2.2 (1.2–3.5) IQR	>2.7
	125 critical patients with 78 being septic and 47 non-septic			
	65 healthy controls			

## Data Availability

All relevant data is included in the manuscript.
